# Lifestyle correlates of overweight in adults: a hierarchical approach (the SPOTLIGHT project)

**DOI:** 10.1186/s12966-016-0439-x

**Published:** 2016-11-03

**Authors:** Célina Roda, Hélène Charreire, Thierry Feuillet, Joreintje D. Mackenbach, Sofie Compernolle, Ketevan Glonti, Helga Bárdos, Harry Rutter, Martin McKee, Johannes Brug, Ilse De Bourdeaudhuij, Jeroen Lakerveld, Jean-Michel Oppert

**Affiliations:** 1 Équipe de Recherche en Épidémiologie Nutritionnelle (EREN), Université Paris 13, Centre de Recherche en Épidémiologie et Statistiques, Inserm (U1153), Inra (U1125), Cnam, COMUE Sorbonne Paris Cité, Bobigny, F-93017 France; 2 Université Paris-Est, Lab-Urba, Créteil, France; 3 Department of Epidemiology and Biostatistics, EMGO Institute for Health and Care Research, VU University Medical Center, Amsterdam, The Netherlands; 4 Department of Movement and Sport Sciences, Faculty of Medicine and Health Sciences, Ghent University, Ghent, Belgium; 5 ECOHOST – The Centre for Health and Social Change, London School of Hygiene and Tropical Medicine, London, UK; 6 Department of Preventive Medicine, Faculty of Public Health, University of Debrecen, Debrecen, Hungary; 7 Sorbonne Universités, Université Pierre et Marie Curie, Université Paris 06, Institute of Cardiometabolism and Nutrition, Department of Nutrition, Pitié-Salpêtrière Hospital, Assistance Publique-Hôpitaux de Paris, Paris, France

**Keywords:** CART, Eating habits, Lifestyle-related behaviors, Obesity, Physical activity, Sedentary behavior, Sleep, Smoking status, Television viewing

## Abstract

**Background:**

Obesity-related lifestyle behaviors usually co-exist but few studies have examined their simultaneous relation with body weight. This study aimed to identify the hierarchy of lifestyle-related behaviors associated with being overweight in adults, and to examine subgroups so identified.

**Methods:**

Data were obtained from a cross-sectional survey conducted across 60 urban neighborhoods in 5 European urban regions between February and September 2014. Data on socio-demographics, physical activity, sedentary behaviors, eating habits, smoking, alcohol consumption, and sleep duration were collected by questionnaire. Participants also reported their weight and height. A recursive partitioning tree approach (CART) was applied to identify both main correlates of overweight and lifestyle subgroups.

**Results:**

In 5295 adults, mean (SD) body mass index (BMI) was 25.2 (4.5) kg/m^2^, and 46.0 % were overweight (BMI ≥25 kg/m^2^). CART analysis showed that among all lifestyle-related behaviors examined, the first identified correlate was sitting time while watching television, followed by smoking status. Different combinations of lifestyle-related behaviors (prolonged daily television viewing, former smoking, short sleep, lower vegetable consumption, and lower physical activity) were associated with a higher likelihood of being overweight, revealing 10 subgroups. Members of four subgroups with overweight prevalence >50 % were mainly males, older adults, with lower education, and living in greener neighborhoods with low residential density.

**Conclusion:**

Sedentary behavior while watching television was identified as the most important correlate of being overweight. Delineating the hierarchy of correlates provides a better understanding of lifestyle-related behavior combinations which may assist in targeting preventative strategies aimed at tackling obesity.

## Background

Excess body weight is determined by multiple factors acting in combination, including genetic, metabolic and behavioral factors, as well as more upstream socio-economic influences and built environment characteristics [1]. Those that are modifiable provide important potential targets for preventive interventions [2]. Diet and physical activity are recognized as the most proximal determinants of energy balance [3] but there is growing recognition of the role of sedentary behaviors (e.g., sitting time), independent of physical activity [4–7]. The influences of smoking and alcohol intake on body weight are also well documented [8–10]. More recently, a role has also been suggested for sleep duration [11–13].

The inter-relationship of these obesity-related lifestyle behaviors has stimulated interest in co-occurrence patterns [14, 15]. Several studies have used explorative data-driven methods, such as cluster analysis or latent class analysis to examine the relations between diet, physical activity, and sedentary behaviors, independently of the health outcome of interest [6, 16, 17]. Smoking status and alcohol consumption have been included in some analyses [18–20]. The variety of methodologies used make it difficult to ascertain how these factors correlate with each other and what this means for body weight and health. Additionally, previous studies have not considered contextual factors such as socio-economic characteristics and the built environment, increasingly recognized as major upstream determinants of overweight [21].

A recursive partitioning method—the classification and regression tree (CART) approach [22]—makes it possible to examine how a set of risk factors jointly influence the risk of an outcome such as overweight. This approach has previously been used to assess the risk of overweight in children [23, 24] and the risk of reduced mobility in older obese adults [25].

This study sought to identify the hierarchy of lifestyle-related behaviors associated with overweight in European adults, and to examine how subgroups identified differed by socio-demographic and built environment characteristics.

## Methods

### Study design and sampling

This study, part of the EU-funded SPOTLIGHT project [26], was conducted in five European urban regions: Ghent and suburbs (Belgium), Paris and inner suburbs (France), Budapest and suburbs (Hungary), the Randstad (a conurbation including Amsterdam, Rotterdam, the Hague and Utrecht in the Netherlands) and Greater London (United Kingdom). Sampling of neighborhoods and recruitment of participants have been described in detail elsewhere [27]. Briefly, neighborhood sampling was based on a combination of residential density and socio-economic status (SES) data at the neighborhood level. This resulted in four pre-specified neighborhood types: low SES/low residential density, low SES/high residential density, high SES/low residential density and high SES/high residential density. In each country, three neighborhoods of each neighborhood type were randomly sampled (i.e. 12 neighborhoods per country, 60 neighborhoods in total). Subsequently, adult inhabitants (≥18 years) were invited to participate in a survey. A total of 6037 individuals participated in the study between February and September 2014. The study was approved by the corresponding local ethics committees of participating countries and all participants in the survey provided informed consent.

### Measures

#### Body mass index

Body mass index (BMI) was calculated by dividing self-reported weight (kg) by the square of the self-reported height (m^2^). Adults were categorized as overweight if their BMI was ≥25 kg/m^2^ [1].

#### Socio-demographic data

Socio-demographic variables included age, gender and educational level (defined as ‘lower’ [from less than primary to higher secondary education] and ‘higher’ [college or university level] to allow comparison between country-specific education systems).

#### Physical activity

Physical activity during the last 7 days was documented using questions from the long version of the validated International Physical Activity Questionnaire (IPAQ) [28]. Good reliability (Spearman correlation coefficients ranged from 0.46 to 0.96) and acceptable criterion validity (median ρ of about 0.30) have been found for this questionnaire in a 12 country study [28]. Transport-related and leisure time physical activity were estimated (in minutes per day − min/d) by multiplying the frequency (number of days in the last 7 days) and duration (average time/d).

#### Sedentary behavior

The validated Marshall questionnaire was used to collect sedentary behavior data during the last 7 days [29]. Acceptable criterion validity (Spearman correlation coefficient greater than or equal to 0.50 for watching TV, and using a computer at home during weekdays) has been demonstrated. Lowest validity coefficients were found for other leisure-time activities and transport-related sedentary behaviors during weekend days (correlation coefficients ranged from 0.15 to 0.42) [29]. Time spent (min/d) sedentary for travel, television (TV), computer and other leisure time activities (e.g., socializing, movies but not including TV and computer use) was averaged over a week.

#### Eating habits

Current eating habits were assessed using common food frequency questions on consumption of fruit, vegetables, fish, sweets, fast-food, sugar-sweetened beverages, and alcohol. Response options were ‘once a week or less’, ‘2 times a week’, ‘3 times a week’, ‘4 times a week’, ‘5 times a week’, ‘6 times a week’, ‘7 times a week’, ‘twice a day’, and ‘more than twice a day’.

#### Smoking status

Participants reported their smoking status: current, former or never.

#### Sleep duration

Participants provided information on their hours of sleep during an average night. The response options ranged from 4 to 16 h/night (in half-hour intervals).

#### Neighborhood clusters

Four neighborhood clusters were previously identified based on data related to food and physical activity features of the built environment collected by a Google Street View-based virtual audit performed in 59 study neighborhoods [30]. The clusters were labeled: cluster 1 (*n* = 33) ‘green neighborhoods with low residential density’, cluster 2 (*n* = 16) ‘neighborhoods supportive of active mobility’, cluster 3 (*n* = 7) ‘high residential density neighborhoods with food and recreational facilities’, and cluster 4 (*n* = 3) ‘high residential density neighborhoods with low level of aesthetics’.

### Data analysis

#### CART approach

Recursive partitioning was used to identify the hierarchy and combinations of all lifestyle-related behaviors described in the Measures section that best differentiated overweight (≥25 kg/m^2^) vs. non-overweight (<25 kg/m^2^) participants.

Recursive partitioning is an algorithm of the CART nonparametric statistical method [22]. This approach has been used in different research fields, such as genetic epidemiology [31], and produced greater homogeneity in subgroups than has been achieved with other approaches, such as regression models [32]. Recursive partitioning is a step-by-step process by which a decision tree is built by either splitting or not splitting each node of the tree into two daughter nodes. Each possible split among all variables present at each node is considered. The tree is constructed by the algorithm asking a sequence of hierarchical Boolean (yes/no) questions (e.g., is X_i_ ≤ θ_j_ ?, where X_i_ is a candidate variable, and θ_j_ is a cut-off) generating descendant nodes [33]. The cut-off in the candidate variable that produced the maximal differentiation between individuals is retained, and used to split the sample into two subgroups (i.e. two daughter nodes). This process is repeated for each new subgroup found. Every variable is a potential candidate at each stage in growing the tree, so some variables may appear several times, using different cut-offs. The best way to split the data is determined by the Gini impurity index. This index ranges from 0 (pure node, i.e. all observations within the node assigned to a single target class—e.g., a node with a class distribution [0;1]) to 1 (impure node, i.e. mixed target classes—e.g., a node with a class distribution [0.5;0.5]). The complete tree is pruned by a sequential node-splitting process to avoid over-fitting the data; a sequence of sub-trees is generated and compared. The optimum tree is obtained using both cross-validation and cost-complexity pruning method. The cost-complexity pruning method assesses the balance between misclassification costs and complexity of the sub-tree. Additionally, each terminal node was set to require a minimum of 200 subjects.

#### Lifestyle subgroups

Characteristics of the subgroups identified through the CART analysis were compared. All variables included in the CART analysis were considered, in addition to socio-demographic and built environment characteristics (i.e. urban region, neighborhood type—pre-specified neighborhood type, and residential density and SES levels examined separately—and neighborhood cluster).

Chi-squared tests, and Kruskal-Wallis tests with post-hoc Bonferroni-Dunn test were used to examine differences between subgroups.

#### Multilevel regression analyses

Because participants were nested within neighborhoods, the likelihood of being overweight for each partitioning variable was estimated by a multilevel logistic regression model (neighborhood identifier included as a random effect) adjusted for potential confounders (gender, age, education level, and neighborhood type).

Statistical analyses were performed using R version 3.2 [34] (‘R-part’ package [35]), and STATA software (release 13.0; Stata Corporation, College Station, TX, USA).

## Results

### Characteristics of the study population

Results are given for 5295 individuals for whom BMI was available. The study population comprised 55.8 % females, with a mean (standard deviation-SD) age of 51.7 (16.4) years; 54 % were highly educated. Mean BMI was 25.2 (4.5) kg/m^2^, and 46.0 % adults were overweight. Compared to non-overweight subjects, overweight adults were more likely to be male, older, less educated, former smokers, short sleepers, less physically active, eating less fruit and vegetables, and spending more time sitting, especially when viewing TV. The prevalence of overweight ranged from 38.3 % in Greater Paris to 53.2 % in Greater Budapest (Table 1).

**Table 1:** Characteristics of the overall study population and according to weight status in the SPOTLIGHT study

n (%) or median (IQR)	Overall *N* = 5295 100 %	Non-overweight^a^ *n* = 2862 54.0 %	Overweight^b^ *n* = 2433 46.0 %	*p* ^†^
Socio-demographic characteristics				
Gender, (*n* = 5246)				
Male	2316 (44.2)	1059 (37.3)	1257 (52.2)	*<* ***0.001***
Female	2930 (55.8)	1780 (62.7)	1150 (47.8)	
Age (in years), (*n* = 5256)	52.0 (26.0)	47.0 (27.0)	57.0 (23.0)	*<* ***0.001***
Education, (*n* = 5191)				
High level	2804 (54.0)	1756 (62.4)	1048 (44.1)	*<* ***0.001***
Low level	2387 (46.0)	1058 (37.6)	1329 (55.9)	
BMI (kg/m^2^), (*n* = 5295)	24.6 (5.5)	22.3 (2.9)	27.8 (4.2)	*<* ***0.001***
Lifestyle-related behaviors				
Smoking status, (*n* = 5247)				
Never	3049 (58.1)	1783 (62.8)	1266 (52.6)	*<* ***0.001***
Former	1464 (27.9)	637 (22.5)	827 (34.3)	
Current	734 (14.0)	418 (14.7)	316 (13.1)	
Physical activity				
Transport-related physical activity (min/d), (*n* = 5274)	26.0 (59.0)	27.0 (57.0)	26.0 (61.0)	***0.012***
Leisure-time physical activity (min/d), (*n* = 5274)	26.0 (44.0)	26.0 (44.0)	21.0 (47.0)	*<* ***0.001***
Sedentary behaviors				
Television time (min/d), (*n* = 4481)	137.0 (120.0)	120.0 (120.0)	154.0 (146.0)	*<* ***0.001***
Computer time (min/d), (*n* = 4358)	77.0 (103.0)	77.0 (98.0)	91.0 (108.0)	*<* ***0.001***
Other leisure sitting time (min/d), (*n* = 3942)	64.0 (112.0)	69.0 (112.0)	60.0 (129.0)	*0.064*
Transport-related sitting time (min/d), (*n* = 4100)	60.0 (73.0)	60.0 (71.0)	60.0 (74.0)	*<* ***0.001***
Eating habits				
Fruit intake (times per week), (*n* = 5198)	7.0 (3.0)	7.0 (3.0)	7.0 (3.0)	*<* ***0.001***
Vegetables intake (times per week), (*n* = 5253)	7.0 (2.0)	7.0 (1.0)	7.0 (2.0)	*<* ***0.001***
Fish intake (times per week), (*n* = 5187)	0.5 (1.5)	0.5 (1.5)	0.5 (1.5)	*0.116*
Fast-food intake (times per week), (*n* = 4803)	0.5 (0)	0.5 (0)	0.5 (0)	*0.213*
Sweets intake (times per week), (*n* = 5149)	3.0 (5.5)	3.0 (5.5)	3.0 (4.5)	***0.004***
Sugar-sweetened beverages consumption (times per week), (*n* = 5073)	2.0 (5.5)	2.0 (5.5)	2.0 (6.5)	*0.349*
Alcohol consumption (times per week), (*n* = 5011)	2.0 (5.5)	3.0 (5.5)	2.0 (5.5)	*0.487*
Sleep duration (hours/night), (*n* = 5269)	7.0 (1.5)	7.0 (1.5)	7.0 (2.0)	*<* ***0.001***
Environmental factors				
Urban region, (*n* = 5295)				
Ghent region	1651 (31.2)	850 (29.7)	801 (32.9)	*<* ***0.001***
Greater Paris	737 (13.9)	455 (15.9)	282 (11.6)	
Greater Budapest	824 (15.5)	386 (13.5)	438 (18.0)	
Randstad	1412 (26.7)	804 (28.1)	608 (25.0)	
Greater London	671 (12.7)	367 (12.8)	304 (12.5)	
Neighborhood type, (*n* = 5223)				
HSES/HRD	1269 (24.3)	725 (25.7)	544 (22.5)	*<* ***0.001***
LSES/HRD	1230 (23.5)	635 (22.5)	595 (24.8)	
HSES/LRD	1325 (25.4)	764 (27.1)	561 (23.5)	
LSES/LRD	1399 (26.8)	699 (24.7)	700 (29.2)	
Neighborhood cluster, (*n* = 4618)				
Green neighborhood with LRD	3022 (65.6)	1588 (63.2)	1434 (68.1)	***0.001***
Neighborhood supportive of active mobility	1150 (24.9)	648 (25.8)	502 (23.9)	
HRD neighborhood with food and recreational facilities	265 (5.7)	162 (6.4)	103 (4.9)	
HRD neighborhood with low level of aesthetics	181 (3.9)	115 (4.6)	66 (3.1)	

*Abbreviations*: *BMI* body mass index, *H*- high-, *IQR* interquartile range, *L*- low-, *RD* residential density, *SD* standard deviation, *SES* socio-economic status

^a^Non-overweight: BMI <25 kg/m^2^

^b^Overweight: BMI ≥25 kg/m^2^

^†^ *p*-value from Chi-squared or Kruskal-Wallis test comparing overweight and non-overweight subjects

Boldface indicates statistical significance

### CART analysis

**Fig. 1:**
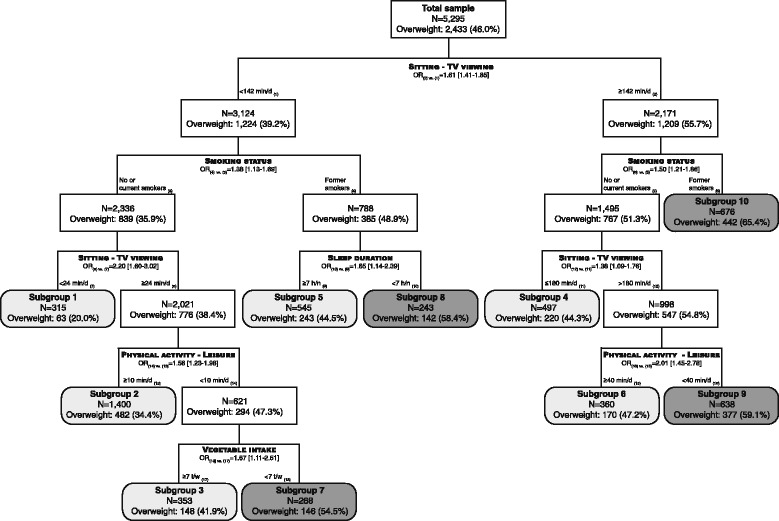
Recursive partitioning analysis (CART) of lifestyle-related behaviors for overweight status in SPOTLIGHT study (*N* = 5295). In dark grey are the identified subgroups with overweight prevalence above 50 %, and in light grey, those with overweight prevalence below 50 %. OR [95 %], odds ratios and confidence intervals at 95 % for each partitioning variable obtained by multilevel logistic regression model (dependent variable: overweight [yes/no], independent variables: partitioning variable identified by CART, gender, age, education, neighborhood type, and neighborhood identifier included as a random effect) are also provided. *Abbreviations*: *h/n* hours per night, *min/d* minutes per day, *t/w* times per week

The final tree contained 10 nodes (i.e. 10 subgroups) and had a classification error of 35.4 %. The 6 variables that were retained as the most important for discriminating overweight status were in the following order: sedentary time while watching TV, smoking status, sleep duration, leisure time physical activity, and vegetable intake (Fig. 1).

The odds of being overweight were 61 % (41–85 %) higher for those reporting longer time watching TV (≥142 min/d) than others.

Longer time spent watching TV (≥142 min/d) and being a former smoker were important correlates of overweight. Current or non-smokers who spent a long time watching TV and were less physically active during leisure time were also at risk of being overweight.

Among adults watching less TV (<142 min/d) and being former smokers, those who were short sleepers (<7 h/night) were more likely to be overweight compared to long sleepers. Protective factors against being overweight among current and non-smokers included: short time watching TV, being physically active during leisure time, and eating vegetables every day.

### Lifestyle subgroups

Table 2 shows the characteristics of the subgroups identified by CART. The proportion of overweight subjects ranged from 20 % (Subgroup 1) to 65.4 % (Subgroup 10). Overall, participants from the various subgroups differed in terms of lifestyle-related behaviors as well as socio-demographic and built environment characteristics.

**Table 2:** Profiles of the subgroups identified by recursive partitioning analysis (CART) in the SPOTLIGHT study

*N* = 5295, n (%) or median (IQR)	Subgroup 1 *n* = 315 (5.9)	Subgroup 2 *n* = 1400 (26.4)	Subgroup 3 *n* = 353 (6.7)	Subgroup 4 *n* = 497 (9.4)	Subgroup 5 *n* = 545 (10.3)	Subgroup 6 *n* = 360 (6.8)	Subgroup 7 *n* = 268 (5.1)	Subgroup 8 *n* = 243 (4.6)	Subgroup 9 *n* = 638 (12.0)	Subgroup 10 *n* = 676 (12.8)	*p* ^†^
Socio-demographic characteristics											
Gender											
Male	133 (42.5)	550 (39.5)	122 (34.8)	201 (40.5)	251 (46.5)	165 (46.3)	121 (45.3)	136 (57.1)	242 (38.7)	395 (59.2)	*<* ***0.001***
Female	180 (57.5)	842 (60.5)	229 (65.2)	295 (59.5)	289 (53.5)	191 (53.7)	146 (54.7)	102 (42.9)	384 (61.3)	272 (40.8)	
Age (in years)	38.0 (20.0)^a^	47.0 (24.0)^a,b^	49.0 (25.5)^a,c^	52.0 (27.0)^a,b,d^	51.5 (25.0)^a,b,e^	60.5 (22.0)^a,b,c,d,e,f^	45.0 (21.0)^a,d,e,f,g^	53.0 (24.0)^a,b,g,h^	57.0 (24.0)^a,b,c,d,g,i^	63.0 (18.0)^a,b,c,d,e,g,h,i^	*<* ***0.001***
Education											
High level	243 (78.4)	893 (65.3)	209 (60.6)	251 (51.3)	344 (63.9)	134 (37.6)	128 (50.2)	139 (58.4)	228 (36.1)	235 (35.5)	*<* ***0.001***
Low level	67 (21.6)	474 (34.7)	136 (39.4)	238 (48.7)	194 (36.1)	222 (62.4)	127 (49.8)	99 (41.6)	403 (63.9)	427 (64.5)	
Overweight	63 (20.0)	482 (34.4)	148 (41.9)	220 (44.3)	243 (44.6)	170 (47.2)	146 (54.5)	142 (58.4)	377 (59.1)	442 (65.4)	*<* ***0.001***
BMI (kg/m^2^)	22.3 (4.0)^a^	23.5 (4.9)^a,b^	24.0 (5.1)^a,c^	24.5 (5.4)^a,b,d^	24.6 (4.9)^a,b,e^	24.7 (5.3)^a,b,f^	25.3 (5.2)^a,b,c,e,g^	25.5 (6.0)^a,b,c,d,e^	25.8 (6.1)^a,b,c,d,e,f,g,h^	26.7 (5.5)^a,b,c,d,e,f,g,h^	*<* ***0.001***
Lifestyle-related behaviors											
Tobacco smoke status											
No smoker	262 (83.4)	1 189 (85.7)	279 (79.7)	403 (81.9)	0	263 (74.7)	194 (74.3)	0	459 (73.2)	0	*<* ***0.001***
Former	0	0	0	0	545 (100)	0	0	243 (100)	0	676 (100)	
Current	52 (16.6)	198 (14.3)	71 (20.3)	89 (18.1)	0	89 (25.3)	67 (25.7)	0	168 (26.8)	0	
Physical activity											
Transport-related physical activity (min/d)	26.0 (53.0)^a^	29.0 (52.0)^b^	13.0 (40.0)^a,b,c^	26.0 (53.0)^c,d^	26.0 (55.0)^c,e^	77.0 (94.0)^a,b,c,d,e,f^	9.0 (30.0)^a,b,d,e,f,g^	20.0 (51.0)^c,f,g,h^	19.0 (51.0)^b,c,f,g,i^	36.0 (93.0)^b,c,e,f,g,h,i^	*<* ***0.001***
Leisure-time physical activity (min/d)	26.0 (40.0)^a^	36.0 (39.0)^a,b^	0 (4.0)^a,b,c^	26.0 (45.0)^b,c,d^	26.0 (42.0)^b,c,e^	86.0 (60.0)^a,b,c,d,e,f^	0 (4.0)^a,b,d,e,f,g^	17.0 (53.0)^b,c,f,g,h^	9.0 (20.0)^a,b,c,d,e,f,g,h,i^	26.0 (54.0)^b,c,d,f,g,i^	*<* ***0.001***
Domain-specific sedentary behaviors											
Television time (min/d)	0 (13.0)^a^	94.0 (60.0)^a,b^	90.0 (60.0)^a,c^	167.0 (26.0)^a,b,c,d^	94.0 (60.0)^a,d,e^	257.0 (120.0)^a,b,c,d,e,f^	94.0 (60.0)^a,d,f,g^	86.5 (64.0)^a,d,f,h^	257.0 (120.0)^a,b,c,d,e,g,h,i^	219.0 (120.0)^a,b,c,d,e,f,g,i^	*<* ***0.001***
Computer time (min/d)	77.0 (136.5)^a^	91.2 (89.3)^a,b^	60.0 (90.0)^a,c^	77.0 (94.0)^b,c,d^	60.0 (81.0)^a,e^	129.0 (14.6)^a,b,c,d,e,f^	77.0 (98.0) ^b,c,e,f,g^	77.0 (73.5)^b,c,e,f,h^	120.0 (146.0)^a,b,c,d,e,g,h^	103.0 (120.0)^a,b,c,d,e,f,g,h^	*<* ***0.001***
Other leisure sitting time (min/d)	77.0 (106.0)^a^	69.0 (93.0)^a^	51.0 (90.0)^a,b,c^	60.0 (129.0)^c,d^	69.0 (94.10)^c,d,e^	94.0 (158.5)^a,b,c,d,e,f^	51.0 (120.0)^a,b,e,f^	60.0 (107.0)^f^	63.0 (146.0)^c,f^	60.0 (146.0)^b,c,f^	*<* ***0.001***
Transport-related sitting time (min/d)	60.0 (76.0)^a^	60.0 (68.0)^b^	50.0 (60.0)^b,c^	60.0 (67.0)^c^	53.0 (62.0)^e^	77.0 (103.0)^a,b,c,d,e^	60.0 (59.0)^e,f^	60.0 (73.0)^c^	61.0 (90.0)^c,d^	93.8 (102.1)^b,c,d,f^	*<* ***0.001***
Eating habits											
Fruit intake (times per week)	7.0 (10.0)^a^	7.0 (3.0)^b^	7.0 (3.0)^c^	7.0 (3.0)^b,d^	7.0 (3.0)^e^	7.0 (3.0)^f^	4.0 (5.0)^a,b,c,d,e,f,g^	7.0 (3.0)^g,h^	6.0 (4.0)^a,b,c,e,f,g,h,i^	7.0 (3.0)^g,i^	*<* ***0.001***
Vegetable intake (times per week)	7.0 (2.0)^a^	7.0 (1.0)^b^	7.0 (0)^a,b,c^	7.0 (2.0)^a,c,d^	7.0 (1.0)^c,e^	7.0 (2.0)^a,b,c,e,f^	5.0 (3.0)^a,b,c,d,e,f,g^	7.0 (2.0)^c,g,h^	7.0 (2.0)^a,b,c,e,g,h,i^	7.0 (2.0)^a,b,c,g,i^	*<* ***0.001***
Fish intake (times per week)	0.5 (1.5)^a^	0.5 (1.5)^b^	0.5 (1.5)^c^	0.5 (1.5)^d^	0.5 (1.5)^e^	0.5 (1.5)^f^	0.5 (0)^a,b,c,d,e,f,g^	0.5 (1.5)^g^	0.5 (1.5)^b,f,g^	0.5 (1.5)^g^	*<* ***0.001***
Fast-food intake (times per week)	0.5 (0)	0.5 (0)^a^	0.5 (0)^b^	0.5 (0)^c^	0.5 (0)^d^	0.5 (0)	0.5 (0)^a,b,c,d^	0.5 (0)^e^	0.5 (0)	0.5 (0)^e^	***0.001***
Sweets intake (times per week)	3.0 (5.5)	3.0 (5.5)	3.0 (6.5)	3.0 (4.5)	3.0 (4.5)^a^	3.0 (5.5)	3.0 (4.5)	3.0 (5.5)	3.0 (4.5)^a,b^	3.0 (5.5)^b^	***0.038***
Sugar-sweetened beverages consumption (times per week)	0.5 (4.5)^a^	2.0 (4.5)^b^	2.0 (6.5)^c^	2.0 (6.5)^a,d^	0.5 (3.5)^c,d,e^	2.0 (6.5)^e^	2.0 (6.5)^a,e,f^	2.0 (4.5)^f,g^	3.0 (6.5)^a,b, d,e,g,h^	2.0 (6.5)^h^	*<* ***0.001***
Alcohol consumption (times per week)	2.0 (4.5)^a^	2.0 (4.5)^b^	2.0 (4.5)^c^	2.0 (4.5)^d^	4.0 (6.5)^a,b,c,d,e^	2.5 (5.5)^d^	0.5 (4.5)^e,f^	4.0 (6.5)^a,b,c,d^	2.0 (5.5)^e,g^	3.0 (6.5)^a,b,c,d,f^	*<* ***0.001***
Sleep (hours/night)	7.0 (1.0)^a^	7.0 (1.5)^b^	7.0 (1.5)^c^	7.0 (1.5)^d^	7.5 (1.0)^a,b,c,d,e^	7.0 (2.0)^e,f^	7.0 (1.5)^e,g^	6.0 (0.5)^a,b,c,d,e,f,g,h^	7.0 (2.0)^e,h^	7.0 (2.0)^e,h^	*<* ***0.001***
Environmental factors											
Urban region											
Ghent region	62 (19.7)	364 (26.0)	170 (48.2)	150 (30.2)	148 (27.2)	137 (38.1)	71 (26.5)	57 (23.4)	256 (40.1)	236 (34.9)	*<* ***0.001***
Greater Paris	67 (21.3)	231 (16.5)	59 (16.7)	64 (12.9)	86 (15.8)	25 (6.9)	40 (14.9)	42 (17.3)	63 (9.9)	60 (8.9)	
Greater Budapest	90 (28.6)	219 (15.6)	27 (7.6)	77 (15.5)	95 (17.4)	44 (12.2)	66 (24.6)	50 (20.6)	65 (10.2)	91 (13.4)	
Randstad	62 (19.6)	393 (28.1)	70 (19.8)	137 (27.5)	153 (28.1)	115 (31.9)	66 (24.6)	62 (25.5)	162 (25.4)	192 (28.4)	
Greater London	34 (10.8)	193 (13.8)	27 (7.7)	69 (13.9)	63 (11.5)	39 (10.9)	25 (9.4)	32 (13.2)	92 (14.4)	97 (14.4)	
Neighborhood type											
HSES/HRD	92 (29.5)	378 (27.3)	86 (24.9)	109 (22.2)	127 (23.4)	86 (24.8)	47 (17.7)	54 (22.3)	142 (22.9)	148 (22.0)	*<* ***0.001***
LSES/HRD	69 (22.1)	305 (22.1)	64 (18.4)	133 (27.0)	114 (21.0)	96 (27.6)	75 (28.3)	50 (20.7)	165 (26.7)	159 (23.6)	
HSES/LRD	72 (23.1)	381 (27.5)	100 (28.8)	118 (24.0)	154 (28.4)	77 (22.2)	54 (20.4)	71 (29.3)	129 (20.8)	169 (25.1)	
LSES/LRD	79 (25.3)	319 (23.1)	97 (28.0)	132 (26.8)	148 (27.2)	88 (25.4)	89 (33.6)	67 (27.7)	183 (29.6)	197 (29.3)	
Residential density neighborhood											
Low	151 (48.4)	700 (50.6)	197 (56.8)	250 (50.8)	302 (55.6)	165 (47.5)	143 (54.0)	138 (57.0)	312 (50.4)	366 (54.4)	*0.054*
High	161 (51.6)	683 (49.4)	150 (43.2)	242 (49.2)	241 (44.4)	182 (52.5)	122 (46.0)	104 (43.0)	307 (49.6)	307 (45.6)	
SES neighborhood											
Low	148 (47.4)	624 (45.1)	161 (46.4)	265 (53.9)	262 (48.2)	184 (53.0)	164 (61.9)	117 (48.3)	348 (56.2)	356 (52.9)	*<* ***0.001***
High	164 (52.6)	759 (54.9)	186 (53.6)	227 (46.1)	281 (51.8)	163 (47.0)	101 (38.1)	125 (51.7)	271 (43.8)	317 (47.1)	
Neighborhood cluster											
Green neighb. with LRD	147 (53.1)	752 (62.1)	224 (70.2)	295 (68.6)	307 (63.7)	215 (68.5)	160 (68.7)	128 (62.5)	374 (68.4)	420 (70.1)	*<* ***0.001***
Neighb. supportive of active mobility	80 (28.8)	325 (26.8)	66 (20.7)	94 (21.9)	127 (26.4)	76 (24.2)	52 (22.3)	55 (26.8)	135 (24.7)	140 (23.4)	
HRD neighb. with food and recreational facilities	29 (10.5)	86 (7.1)	9 (2.8)	23 (5.3)	29 (6.0)	17 (5.4)	6 (2.6)	15 (7.3)	26 (4.7)	25 (4.2)	
HRD neighb. with low level of aesthetics	21 (7.6)	49 (4.0)	20 (6.3)	18 (4.2)	19 (3.9)	6 (1.9)	15 (6.4)	7 (3.4)	12 (2.2)	14 (2.3)	

*Abbreviations*: *BMI* body mass index, *H*- high-, *IQR* interquartile range, *L*- low-, *neighb*. neighborhood, *RD* residential density, *SES* socio-economic status

^†^ *p*-value from Chi-square test or Kruskal-Wallis

^a-i^: refer to pairwise comparisons (*p*-value of Dunn’s test with Bonferroni adjustment < 0.05)

Boldface indicates statistical significance

Subgroup 1 (*n* = 315, mean [SD] BMI: 22.7 [3.4] kg/m^2^) consisted of the youngest (40.8 [13.6] years-old), and highly educated participants (78.4 %). This subgroup reported the lowest time spent watching TV (mean [SD]: 5.2 [7.9] min/day, median: 0 min/day), the highest mean frequency of eating fruits and vegetables. The highest percentage of participants living in neighborhoods that were characterized by high SES and high residential density was observed in this subgroup, as was the lowest percentage of participants living in ‘green neighborhoods with low residential density’.

In 4 subgroups (7, 8, 9, and 10), overweight prevalence was >50 %. Members lived mainly in low SES neighborhoods. Subgroup 7 grouped less physically active individuals, who ate fruits, vegetables, and fish less frequently. Subgroup 8 members were short sleepers. The greatest percentage of individuals living in low residential neighborhoods was reported in this subgroup. Subgroup 9 included the greatest percentage of current smokers, individuals who reported long mean time watching TV (mean [SD]: 306.0 [131.3] min/day, median: 257 min/day), and high mean consumption of sugar-sweetened beverages (4.9 [5.7] times/week, median: 3.0 times/week).

Subgroup 10 (*n* = 676, mean [SD] BMI = 27.2 [5.0] kg/m^2^) included mainly males, older (59.6 [14.4] years-old) and low educated adults (64.5 %), who reported high alcohol consumption and living in ‘green neighborhoods with low residential density’.

## Discussion

This study investigated the hierarchy and combination of lifestyle-related behaviors in relation to the prevalence of overweight in European adults. Prolonged sitting while watching TV, being a former smoker, short sleep, lower levels of physical activity and lower vegetable consumption were the lifestyle-behaviors that identified the subgroups with highest likelihood of being overweight. High-risk subgroups included mainly males, older and less well educated adults living in greener neighborhoods with low residential density.

Although it is well recognized that overweight and obesity are multifactorial in origin [1, 2], few studies have examined the joint relation of lifestyle-related behaviors with overweight in adults. In this study, a hierarchy of lifestyle-related behaviors in identifying subgroups at risk was established through a visual chart showing how risk factors are inter-related. The tree indicated that the most important factor was sitting while watching TV. This variable appeared several times at different levels of the tree, underlying its importance. The variable that followed was smoking status, in both tree branches, and no additional variable appeared to explain the risk for overweight in former smokers (among those with longer duration of watching TV), suggesting its very high impact. Sleep duration, leisure time physical activity and vegetable intake appeared at later stages in the tree, suggesting they would have less importance compared to sedentary behavior and smoking status. Relations between the lifestyle-related behaviors and overweight status were confirmed in multilevel regression analyses taking into account potential confounding factors. The findings also suggested nonlinear relations between lifestyle-related behaviors and overweight. Indeed, subgroups who watched TV a lot (>180 min/d) had lower odds of being overweight than subgroups who watched less TV (between 24 min/d and 142 min/d).

Although it has been suggested that a combination of several sedentary behavior variables is appropriate to capture sedentary lifestyle [36], only TV viewing was retained among several variables related to sedentary time. The greater importance of TV viewing has been previously suggested in cross-sectional studies [37–39]. Given the lack of evidence from prospective studies, the issue of bidirectional or reverse causality has been raised [40]. In the Nurses’ Health study, each 2 h/d increment in TV watching was associated with a 23 % [17–30 %] increased risk of obesity. However, the risk of developing obesity was attenuated after adjustment for baseline BMI [5]. These findings may suggest that, even at baseline, women who watched more TV were already on a trajectory to become obese [5]. Heavier individuals at baseline could have a preference for sedentary habits due to their higher body weight. TV viewing is not only an indicator of sedentary behavior but may represent a potential surrogate of other behaviors affecting the energy balance e.g., via increased snacking behavior [7, 41].

Former smokers were more likely to be overweight than both current and never smokers. These results are consistent with previous findings [10, 42–44]. Weight gain after quitting smoking has been related to the fact that nicotine acts as an appetite suppressant and quitting may be associated with increased energy intake [45, 46]. The average weight gain is about 4.5 kg, 1 year after quitting [46]. In the NHANES survey, weight gained over 10-years was significantly higher in former smokers compared to current smokers (8.4 kg vs. 3.5 kg, after adjustment for age, gender, ethnicity, education level) [44]. A recent study has estimated that smoking cessation leads to an average increase of 1.5–1.7 BMI units and that the drop in smoking may explain up to 14 % of the rise in obesity prevalence in recent decades [47]. Weight gain after smoking cessation was less pronounced when number of years since smoking cessation increased [43], and negatively associated with socio-economic status [48].

Short sleep duration was found to be associated with an increased risk of overweight. The hypothesized underlying mechanisms include thermoregulation, hunger hormone regulation changes, and/or an impact on physical activity and sedentary behaviors [49–52]. Short sleep duration was associated with other lifestyle-related behaviors, such as TV or computer use [52, 53], a correlation between time spent sleeping, physical activity and sedentary behavior was documented [54]. High leisure time physical activity and intake of vegetables were associated with lower prevalence of overweight. These behaviors—which tend to co-occur—are both well recognized as healthy lifestyle behaviors [55, 56]. Interestingly, some cut-offs found are close to thresholds previously reported and/or recommended guidelines (e.g., 2 h/d watching TV [5, 38], 7–9 h of sleep [57]). In addition, at least one variable from each component of lifestyle (physical activity, sedentary behavior, sleep duration, eating habits, and smoking status) was identified as a correlate of overweight. Moreover, subgroups at high-risk of overweight were characterized by at least one unhealthy lifestyle behavior. These findings emphasize how all components of lifestyle are important to consider and a combination of unfavorable lifestyle factors may predict overweight in adults.

Lifestyle subgroups identified by CART differed in terms of socio-demographic factors. The subgroup with the highest prevalence of overweight comprised mainly males, older adults and lower educated adults. These findings are in line with previous studies [58, 59]. Individuals with higher educational background may be more informed about the health consequences of their lifestyles and have the resources to take action, leading to healthier lifestyle behaviors [60]. The subgroups identified also varied across urban regions: 72.2 % of French respondents were in the subgroups with lower overweight prevalence (subgroups 1–6). Looking at differences at neighborhood level, as previously documented [61], some neighborhoods seem more obesogenic than others, especially low SES and low residential density neighborhoods. Socio-spatial disparities in obesity prevalence at census-tract level have been previously documented with lower prevalence in neighborhoods with high median home values [61]. Low SES neighborhoods have been shown to have less supportive environmental conditions for active transportation [21]. Moreover, a greater percentage of ‘green neighborhoods with low residential density’ was observed in subgroups with high overweight prevalence. Greener neighborhoods with low residential density may be less supportive of active transport and more oriented towards motorized transport. Use of motorized transportation may be linked to weight gain [62]. Conversely, in high residential density neighborhoods, many destinations are easily accessed since located at shorter distance, and parking a car may be more difficult therefore encouraging active transportation (e.g., walking, cycling, public transport) [21]. Thus, adults living in neighborhoods unsupportive of physical activity and far away from destinations may be more likely to remain indoors and watch TV.

This study has several strengths: a relatively large sample size, assessment of a number of lifestyle-related variables using standardized procedures, a survey performed in different geographical areas across Europe, and the use of a nonparametric method (CART) providing a visual representation of lifestyle-related behavior inter-relationships. This study has some limitations, caution is thus needed when interpreting and generalizing the results. Due to its cross-sectional nature, temporal relations between overweight and lifestyle behaviors cannot be assessed. As data were self-reported, potential (recall) bias, and possible underestimation or overestimation of variables (e.g., weight/height [63], sedentary behaviors [29, 64], physical activity [64–66]) cannot be excluded. Although behaviors such as eating habits were not recorded in enough detail to assess the role of more detailed dietary aspects, such as macronutrient intake, many aspects of lifestyle currently thought to be associated with body weight were covered (sedentary behavior, physical activity, eating habits, alcohol consumption, smoking status, and sleep duration). The CART method is data-driven, and the misclassification error was about 35 %. In the literature, it is not uncommon to report a misclassification error around 30 % and this might be higher for health promotion-based intervention strategies [67].

## Conclusions

Low levels of TV viewing, non-smoking, high leisure time physical activity, high vegetable consumption, and longer sleep duration were identified as components of a healthy lifestyle associated with decreased risk of excess weight in adults. The results specifically point to the importance of sedentary habits as a key component to focus on when addressing the multiple factors associated with excess weight in preventive interventions.

## Abbreviations

BMI: Body mass index
CART: Classification and regression tree
SD: Standard deviation
SES: Socio-economic status
TV: Television
